# Differential Gene Expression Segregates Cattle Confirmed Positive for Bovine Tuberculosis from Antemortem Tuberculosis Test-False Positive Cattle Originating from Herds Free of Bovine Tuberculosis

**DOI:** 10.1155/2012/192926

**Published:** 2012-06-04

**Authors:** Ailam Lim, Juan P. Steibel, Paul M. Coussens, Daniel L. Grooms, Steven R. Bolin

**Affiliations:** ^1^Department of Pathobiology and Diagnostic Investigation, Michigan State University, East Lansing, MI 48824, USA; ^2^Diagnostic Center for Population and Animal Health, Michigan State University, 4125 Beaumont Road, Lansing, MI 48910, USA; ^3^Department of Animal Science, Michigan State University, East Lansing, MI 48824, USA; ^4^Department of Fisheries and Wildlife, Michigan State University, East Lansing, MI 48824, USA; ^5^Department of Large Animal Clinical Sciences, Michigan State University, East Lansing, MI 48824, USA

## Abstract

Antemortem tests for bovine tuberculosis (bTB) currently used in the US measure cell-mediated immune responses against *Mycobacterium bovis*. Postmortem tests for bTB rely on observation of gross and histologic lesions of bTB, followed by bacterial isolation or molecular diagnostics. Cumulative data from the state of Michigan indicates that 98 to 99% of cattle that react positively in antemortem tests are not confirmed positive for bTB at postmortem examination. Understanding the fundamental differences in gene regulation between antemortem test-false positive cattle and cattle that have bTB may allow identification of molecular markers that can be exploited to better separate infected from noninfected cattle. An immunospecific cDNA microarray was used to identify altered gene expression (*P* ≤ 0.01) of 122 gene features between antemortem test-false positive cattle and bTB-infected cattle following a 4-hour stimulation of whole blood with tuberculin. Further analysis using quantitative real-time PCR assays validated altered expression of 8 genes that had differential power (adj  *P* ≤ 0.05) to segregate cattle confirmed positive for bovine tuberculosis from antemortem tuberculosis test-false positive cattle originating from herds free of bovine tuberculosis.

## 1. Introduction

 Bovine tuberculosis (bTB) caused by *Mycobacterium bovis *(*M. bovis*) occurs worldwide and has been estimated to cause annual losses of three billion dollars to global agriculture [1, 2]. In addition to being an important pathogen of cattle, *M. bovis* may infect many other domestic and wildlife species, and it infects humans [3–5]. The zoonotic aspect of bTB is underappreciated, as documented by The World Health Organization recently listing bTB as a neglected zoonotic disease [6]. Thus, control of bTB is a continuing effort that is necessary to protect livestock, wildlife, and human populations. In many developed countries, control of bTB is based on “test and slaughter” programs. Field and/or laboratory diagnostic tests are used to identify potentially infected cattle herds for quarantine, which may be followed by additional diagnostic testing and slaughter of all cattle that show positive test reactions. Although proven effective, “test and slaughter” programs are costly and have not been adopted by most developing countries.

 The bTB control program in the United States (US) has reduced the prevalence of bTB-infected cattle herds from an estimated 5% of all herds in 1917 to an estimated infection rate of <0.001% for all herds [7]. Most antemortem diagnostic tests currently approved in many countries for detection of bTB measure cell-mediated immune responses. In the US, approved tests include the caudal fold tuberculin skin test (CFT), as the primary screening test, and either the comparative cervical tuberculin skin test (CCT) or the whole blood interferon-gamma (IFN-*γ*) ELISA assay as secondary tests. Cattle that show positive reactions in successive primary and secondary tests usually are culled for postmortem examination. Predictably, as prevalence of bTB-infected cattle decreases, the proportion of antemortem test-false positive cattle culled for postmortem examination increases [8]. The prevalence of bTB in the state of Michigan is low, and only 1-2% of cattle that show positive responses on two successive antemortem tests are confirmed as positive for bTB at postmortem examination [9]. Thus, there is a need for ancillary antemortem tests for bTB that improve the positive predictive value of the testing process.

 DNA microarray technologies facilitate rapid and large-scale examination of global gene expression profiles. This allows efficient detection of target genes that might be affected by a treatment or a disease process. This approach for identification of altered gene expression is particularly useful in studies of host response to various infections [10–13]. Altered gene expression profiles may show common patterns of response across different host cell types with different pathogens [10, 11]. Also, unique gene expression signatures can be identified in a host's response to a specific pathogen. This may be attributed to pathogen-driven differences in reprogramming of host gene transcription at the host-pathogen interface [12–14]. Thus, mining differential host transcriptome response to identify molecular events associated with pathogenesis offers an opportunity for discovery of diagnostic molecular markers predictive for specific infectious, metabolic, or genetic diseases [15–18]. Recently, microarray platforms of bovine genes have been used to study pathogenic processes and to identify molecular markers of infection, for two mycobacterial pathogens of cattle; *M. avium *subspecies* paratuberculosis* (MAP) and* M. bovis *[15, 16, 19–24].

Peripheral blood transcriptome profiles are particularly useful for identification of pathogen-associated immune response signatures, which can be used to develop diagnostic tools [10, 12, 14, 23]. Previous studies of bTB by Meade et al. [16, 21, 22] compared peripheral blood mononuclear cells (PBMCs) of cattle that had bTB with normal healthy cattle that tested negative for bTB. In the current study, we compared differential gene expression in cattle that had bTB with cattle that were single or double antemortem test-false positive for bTB. The intent was to identify differential gene expression profiles among cattle that have similar reactions in antemortem bTB tests but differ in their infection status at postmortem examination. Using this approach is critical because cattle that are antemortem test-false positive for bTB currently are not differentiated from truly infected cattle until a time-consuming and expensive postmortem diagnostic process is completed. Our overall objective was to identify gene targets that can be used for differentiation of antemortem test-false positive cattle from cattle that have bTB.

## 2. Materials and Methods

### 2.1. Experimental Animals and bTB Infection Status

Cattle used in this study were submitted for postmortem examination because of positive reactions in antemortem diagnostic tests for bTB. Cattle were transported to the Diagnostic Center for Population and Animal Health (DCPAH) at Michigan State University (MSU) the day before humane euthanasia and postmortem examination were performed. A presumptive positive or negative diagnosis was made at the DCPAH based on the presence of gross and/or microscopic lesions. Regardless of the presumptive diagnosis, samples from all cattle examined were submitted to United States Department of Agriculture's National Veterinary Services Laboratories (NVSLs). Final diagnosis was made by the NVSL based on results of polymerase chain reaction (PCR) assays and/or mycobacterial culture.

Three study groups of cattle were used for microarray analysis. The study groups included bTB positive cattle (bTB, *n* = 4), which were positive for lesions of bTB at postmortem examination and confirmed positive by the NVSL; double antemortem test-false positive cattle (DFP, *n* = 4), which showed positive reactions on primary and secondary diagnostic tests for bTB but were negative for lesions of bTB at postmortem examination, and were confirmed negative for bTB by the NVSL; and single antemortem test-false positive cattle (SFP, *n* = 7), which were positive on the CFT but were negative on the CCT or IFN-*γ* assay, were found negative for lesions of bTB at postmortem examination and were confirmed negative for bTB by the NVSL. The number of cattle in each group was expanded to 10 for validation of altered gene expression, using quantitative real-time PCR (qPCR) assays. Finally, healthy cattle from long-term bTB negative farms (*n* = 12) with recent negative test records for bTB, MAP, bovine leukosis virus, and bovine viral diarrhea virus were used as blood donors to obtain a reference pool of control RNA for the study.

### 2.2. Blood Collection, Antigen Stimulation, and RNA Extraction

Blood (~45 mL) was collected from each animal in the bTB, DFP, and SFP groups into 10 mL heparin-containing evacuated tubes (Vacutainer, BD Diagnostics, Franklin Lakes, NJ) immediately before euthanasia for postmortem examination. Within 3 hours of collection, the blood from each animal was pooled into individual sterile 50 mL conical tubes and stimulated with purified protein derivative prepared from heat-killed cultures of *M. bovis* (bPPD) (Prionics AG, Switzerland) at 20 *μ*g bPPD/mL of blood. The blood was incubated at 38 ± 1°C for 4-hours prior to harvest. Blood samples from the 12 healthy cattle were similarly collected, processed, and stimulated. The 4 hour period for antigen stimulation was chosen because it was considered the maximal time that could be used if receipt of sample, stimulation of blood, and extraction of RNA were to occur during a 10-hour diagnostic laboratory work day.

After stimulation, the blood was centrifuged at 1200 ×g for 15 minutes at 18°C to form layers of plasma, buffy coat cells and red blood cells. The buffy coat layer of cells, and 2 mL of red blood cells immediately below the buffy coat layer were harvested by aspiration and transferred to a new 50 mL conical tube. Two rounds of hypotonic lysis of red blood cells were performed by addition of ice-cold diethylpyrocarbonate (DEPC) treated-sterile deionized water for 2 minutes, followed by addition of an equal volume of ice-cold DEPC-treated sterile 2X saline (1.7% w/v NaCl). Intact cells were pelleted by centrifugation at 1200 ×g for 15 minutes at 18°C after the first round of hypotonic lysis, then at 190 ×g for 10 minutes at 4°C after the second round. After the second round of hypotonic lysis, the supernatant was decanted, and 1 mL TRIzol Reagent (Invitrogen, Carlsbad, CA) was added to the loose cell pellet for each 9 mL beginning volume of whole blood. This mixture was frozen at −84°C until use. For isolation of RNA, the mixture was thawed on ice and subjected to 10 passages through a 20-gauge needle. The resulting homogenate was divided into 1 mL aliquots, and the remainder of the RNA extraction procedure was performed according to the manufacturer's recommendations. The total cellular RNA from each animal was then pooled into a single tube and treated with RQ1 RNase-Free DNase (Promega, Madison, WI) according to manufacturer's recommendations. The treated RNA was extracted again using equal volumes of phenol-chloroform, followed by purification using MEGAclear Purification Kit (Ambion, Austin, TX). The purified RNA was immediately stored at −84°C until use.

Before use, the RNA from the study cattle was thawed on ice, and the integrity and concentration of the RNA was determined using the Agilent 2100 Bioanalyzer and RNA Nano 6000 Kit (Agilent Technologies, Santa Clara, CA). The RNA from the 12 healthy cattle was mixed to form a homogenous control reference pool and the integrity and concentration of that pooled RNA was similarly determined.

### 2.3. Experimental Design, cDNA Synthesis, and Microarray Hybridization

The BOTL-5 cDNA microarray used in this study was the 5th generation of a previously described bovine total leukocyte immunospecific microarray [25, 26]. The gene content and sequence information for BOTL-5 microarray are available at the National Center for Biotechnology Information Gene Expression Omnibus (NCBI GEO, platform number GPL5751). Briefly, BOTL-5 contains 3,888 features including 1,391 bovine expressed sequence tag (EST) cloned inserts and PCR amplicons derived from known sequences of bovine immune response genes, spotted in duplicate along with multiple replicates of microarray specific control features. A common reference design was used for microarray hybridization in this study. That design was selected because the cattle used were submitted for postmortem examination over a two-year period. The use of a common reference on each microarray allowed comparison of gene expression of individual samples obtained over a large time span, and allowed comparison of gene expression across the various study groups [27–29].

The cDNA synthesis and dye labeling were performed with 15 *μ*g aliquots of total RNA using the SuperScript III Fluorescent Labeling Kit containing Cy5 and Cy3 dyes (Cat no. L101401; Invitrogen Corp., Carlsbad, CA) following the manufacturer's recommendations. RNA from the study cattle was labeled with the Cy3 dye and cohybridized with the control reference pool of RNA labeled with Cy5 dye. For each microarray experiment, the Cy3-labeled sample and Cy5-labeled reference pool were combined and concentrated, using a Microcon 30 centrifugal filter unit (Millipore, Billerica, MA). The labeled cDNA mixture was eluted in 110 *μ*L of SlideHyb buffer no. 3 (Ambion, Austin, TX) and heated for 5 minutes at 70°C prior to hybridization. The hybridization was done using a GeneTAC HybStation (Genomic Solutions Inc., Ann Arbor, MI) and an 18-hour step-down protocol (3 hours at 60°C, 3 hours at 55°C, 12 hours at 50°C). Immediately following hybridizations, the slides were subjected to 5 washes of 30 seconds each at 50°C with 2x SSC containing 0.1% SDS, 5 washes of 30 seconds each at 42°C with 0.2x SSC containing 0.1% SDS and 5 washes of 30 seconds each at 42°C with 0.2x SSC. After removal from the hybridization unit, the microarray slides were rinsed once in 2x SSC and once in double-distilled water and then dried by centrifugation for two minutes at 1,200 ×g. Hybridized cDNA microarrays were scanned immediately using a GeneTAC LS IV microarray scanner and GeneTAC LS software (Genomic Solutions Inc., Ann Arbor, MI).

### 2.4. Data Processing, Normalization, and Analysis

 Microarray images were processed using GenePix Pro 6.0 software (Molecular Devices, Downingtown, PA) to generate spot intensity files. The output files were analyzed using the LIMMA (LInear Models for MicroArray) software package [30] implemented in the R language and environment (http://www.r-project.org/) [31]. Briefly, background correction [32] and within microarray normalization [33] were performed prior to linear regression analysis. Prior to and after normalization, MA plots of data were generated for visual assessment of the normalization effect. Log-ratios of median fluorescence intensities were used for data analysis. The empirical Bayes moderated *T* statistic [34] was used to verify altered expression of gene features within each group of cattle (SFP, DFP, and bTB) and between groups of cattle (bTB versus SFP, bTB versus DFP, and DFP versus SFP).

### 2.5. Quantitative Real-Time PCR (qPCR) Validation of Differential Gene Expression

Twelve potential reference genes were evaluated for stability of expression level within and between the study groups of cattle, using 3 available programs (*BestKeeper, NormFinder, *and* geNorm*) [35]. Succinate dehydrogenase complex subunit A (SDHA) was determined to be the most suitable reference gene for this study (data not shown). The list of 33 genes selected for validation of expression using qPCR assay, PCR primer sequences, primer concentration, PCR efficiency, and amplicon size are given in Table 1. The PCR primers for the gene targets were designed using Clone Manager Suite 7.0 (Sci-Ed Software, Cary, NC) or Primer Express 3.0 software (Applied Biosystem, Foster City, CA) and were synthesized by Integrated DNA Technologies (Coralville, IA). All primers were tested for amplification with the control reference pool of RNA and with a no template control (NTC). Optimal primer concentration for qPCR was empirically determined (data not shown).

Validation of altered gene expression using qPCR was done for 17 gene features selected from the microarray data. Those gene features showed substantial altered expression among cattle or showed unique regulation within a group of cattle (Table 1). An additional 16 genes coding for cytokines or chemokines were selected from the literature for qPCR analysis (Table 1). The cytokines or chemokines produced by those genes are reported as important mediators in bovine and human TB infections. Samples of RNA from 30 cattle (10 cattle per study group) were used to assess altered expression of the 33 selected genes. The samples of RNA subjected for qPCR included some of the original samples used in the microarray experiments plus new samples of RNA from additional cattle that met the criteria for each study group. Synthesis of cDNA was performed with 2 *μ*g of total RNA from each study animal and from the common reference pool of RNA, using commercially available reagents (Superscript II Reverse Transcriptase and Oligo (dT)_12–18_ Primer, Invitrogen, Carlsbad, CA) and the manufacturer's recommended protocol. Upon completion of cDNA synthesis, the RNA template in each reaction was removed with 1U of RNase H (Invitrogen, Carlsbad, CA). The cDNA was purified using QuickClean enzyme Removal Resin (Clontech Laboratories, Mountain View, CA). Finally, the concentration of purified cDNA was measured by spectrometry (ND-1000, NanoDrop Technologies, Wilmington, DE) and diluted to final concentration of 10 ng/*μ*L. The cDNA was stored at −20°C until use in qPCR assays.

The qPCR assays were performed in triplicate using SYBR Green PCR Master Mix and an ABI 7500 Real-time PCR System (Applied Biosystems, Foster City, CA). Each 20 *μ*L reaction consisted of 1x SYBR Green PCR master mix, 30 ng of cDNA, and a pair of primers at predetermined optimal concentrations (Table 1). The reaction conditions were 95°C for 10 minutes, then 40 cycles of 95°C for 15 seconds, and 58°C for 1 minute. Dissociation curve analysis was done for each reaction.

### 2.6. PCR Efficiency Determination and qPCR Data Analysis

The magnitude of the normalized reporter signal (delta Rn) data and the cycle threshold (Ct) data exported from the ABI 7500 SDS software were used to verify that acceptable PCR efficiency (>1.8) was achieved and to calculate the relative expression level of the targeted genes, respectively. The efficiency of each qPCR reaction was determined based on the slope of the exponential phase of the reaction, using the LinRegPCR program [36]. The mathematical model for calculation of relative gene expression proposed by Pfaffl et al. was used for qPCR analysis [37]. The SDHA gene was used as the reference/normalizer gene, and the common reference pool (as in the microarray experiments) was used as the calibrator. For each animal, the mean Ct value from triplicate reactions for each gene target was used to determine the relative gene expression value. To calculate the mean differential expression of a gene target for an entire study group, the relative gene expression values of the gene target for each animal within the group were averaged. Thus, the calculated mean differential expression represents altered expression of a gene target among cattle in a study group relative to the common reference pool of RNA. The Student's *t*-test was used to determine the statistical significance of altered expression of gene targets within each study group (SFP, DFP, and bTB).

The relative gene expression values of each gene target for each animal in a group were used to determine the significance of differential expression of a gene target among groups of cattle (bTB versus SFP, bTB versus DFP, and DFP versus SFP). The analysis was performed with the ANOVA test based on linear fixed effect models [38], implemented in the MAANOVA software package [39]. Simultaneous fitting of multiple linear models was done with 5000 permutation tests and with the jsFDR method for false discovery rate (FDR) adjustment [40].

## 3. Results

### 3.1. Identification of Altered Gene Expression Profiles from Microarray Data

**Figure 1:**
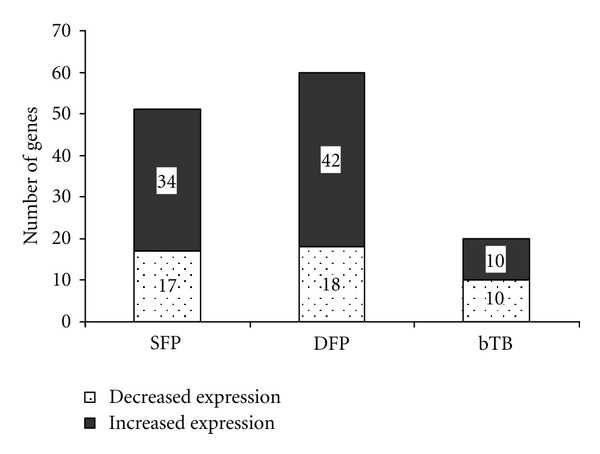
Number of genes from microarray analysis that were differentially expressed (*P* ≤ 0.01) within single antemortem test-false positive (SFP), double antemortem test-false positive (DFP), and bTB-infected (bTB) groups of cattle. The RNA used for microarray analysis was harvested after a 4-hour stimulation of whole blood with tuberculin, and comparison of gene expression levels was with a reference pool of mRNA harvested from the blood of healthy cattle after 4-hour stimulation with tuberculin. The number of genes for each group of cattle that showed increased expression (solid box) or decreased expression (shaded box) relative to the reference pool of RNA is indicated by the figure in the boxes.

A total of 1,391 gene features on the BOTL-5 microarray were analyzed, of which, 122 gene features were differentially expressed (*P* ≤ 0.01) in one or more groups of cattle. Only 9 of the 122 gene features were shared by two groups of cattle; the remaining 113 genes were uniquely regulated within individual groups of cattle (SFP, DFP, and bTB). Overall, we found more genes with altered expression in each group of antemortem test-false positive cattle than in the bTB positive cattle. In both the SFP and DFP groups of cattle, the ratio of gene features showing increased expression levels to those showing decreased expression levels was at least 2 : 1. In contrast, the ratio of increased expression to decreased expression was 1 : 1 in the bTB group of cattle (Figure 1). The complete list of differentially expressed gene is provided in Supplementary Table S1 (see Table S1 in Supplementary Material available online at doi: 10.1155/2012/192926).

**Figure 2:**
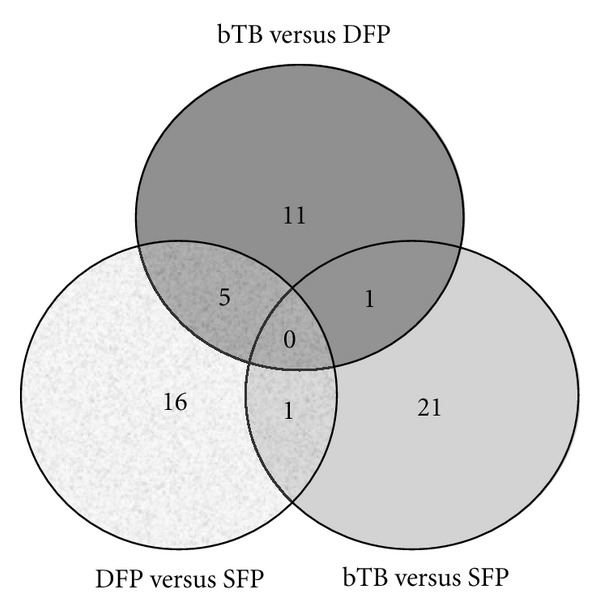
Numbers of gene features identified from analysis of microarray data that were differentially expressed (*P* ≤ 0.01) among single antemortem test-false positive (SFP), double antemortem test-false positive (DFP), and bTB-infected (bTB) groups of cattle. The numbers of genes common to or unique for the groups of cattle are shown in a Venn diagram.

The objective of this study was to find molecular markers that can differentiate antemortem test-false positive cattle from bTB infected cattle. When a comparison of gene expression data was done between groups of cattle, only 55 gene features showed significant statistical power to differentiate the study groups from each other (bTB versus SFP, bTB versus DFP, and DFP versus SFP). The complete list of those genes is provided in Supplementary Table S2. Using the data generated from analysis of the microarrays, differentiation of a particular group of cattle from each of the other two groups was possible, but only a few gene features were useful for that purpose (Figure 2). The DFP group of cattle could be differentiated from the bTB and the SFP groups using the altered expression levels of 5 gene features. Those genes were thioredoxin-related transmembrane protein 4 (TMX4); transmembrane protein, adipocyte associated 1 (TPRA1); major histocompatibility complex, class II, DM alpha-chain (BOLA-DMA); Fc fragment of IgG, receptor transporter alpha (FCGRT); ribosomal protein L19 (RPL19). The altered expression level of only one gene feature, tripartite motif-containing 13 (TRIM13), was useful for differentiating the SFP group of cattle from the bTB and DFP groups. Similarly, altered expression of only one gene feature, clone BOTL0100011_A05 (a gene feature of unknown function), was useful for differentiation of the bTB group of cattle from the SFP and DFP groups.

### 3.2. Group Level Gene Expression Profiling with Quantitative Real-Time PCR (qPCR) Data

**Figure 3:**
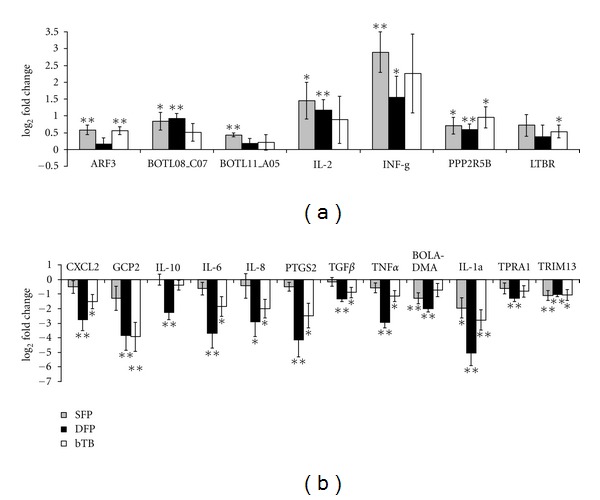
The relative gene expression levels compared with the reference pool of RNA from healthy cattle, as determined by qPCR assays, for the single antemortem test-false positive (SFP, shaded box), double antemortem test-false positive (DFP, solid box), and bTB-infected (bTB, clear box) groups of cattle. Gene expression levels (in log_2_ fold change) were calculated using the published mathematical algorithm [37] in which the reference pool of RNA was set as baseline (0 value at *Y*-axis) and used as the calibrator. Statistically significant differences were determined using Student's *t*-test and are shown at *P* ≤ 0.05 (*) and *P* ≤ 0.01 (**). The error bars represent the standard error of the mean expression level for a group of cattle. (a) Genes with increased expression in all groups of cattle. (b) Genes with decreased expression in all groups of cattle.

With the extended panel of 10 cattle per study group, the statistical significance of the mean altered gene expression within each group of cattle could be assessed with greater accuracy. The qPCR assays identified many genes that showed considerable variation in expression level among cattle within all study groups. Seven genes showed increased expression in all 3 study groups, and 12 genes showed decreased expression in all 3 study groups (Figures 3(a) and 3(b)). The gamma interferon (IFN-*γ*) gene showed the greatest increase in expression in all study groups (2.92- to 7.42-fold). Interleukin-2 (IL-2) also showed a marked increase in expression (1.84- to 2.74-fold). Other genes that showed increased expression in all groups of cattle were serine/threonine-protein phosphatase 2A 56 kDa regulatory subunit beta isoform (PPP2R5B), lymphotoxin beta receptor (LTBR), ADP-ribosylation factor 3 (ARF3), and 2 clones with unknown function (BOTL0100008_C07 and BOTL0100011_A05).

Proinflammatory cytokines were among the downregulated genes, including interleukin-6 (IL-6), tumor necrosis factor alpha (TNF-*α*), and interleukin-1 alpha (IL-1*α*). Also downregulated were anti-inflammatory cytokines, including interleukin-10 (IL-10) and transforming growth factor beta (TGF-*β*), along with several chemokines, including interleukin-8 (IL-8), chemokine (C-X-C motif) ligand 2 (CXCL-2), and chemokine (C-X-C motif) ligand 6 (GCP2). Decreased expression of many of those genes was especially evident in cattle from the DFP group. Other genes that showed decreased expression included the major histocompatibility complex Class II molecule (BOLA-DMA), prostaglandin-endoperoxide synthase 2 (PTGS2), tripartite motif-containing 13 (TRIM13) and transmembrane protein, adipocyte associated 1 (TPRA1).

### 3.3. Analysis of Altered Gene Expression in Individual Cattle

**Figure 4:**
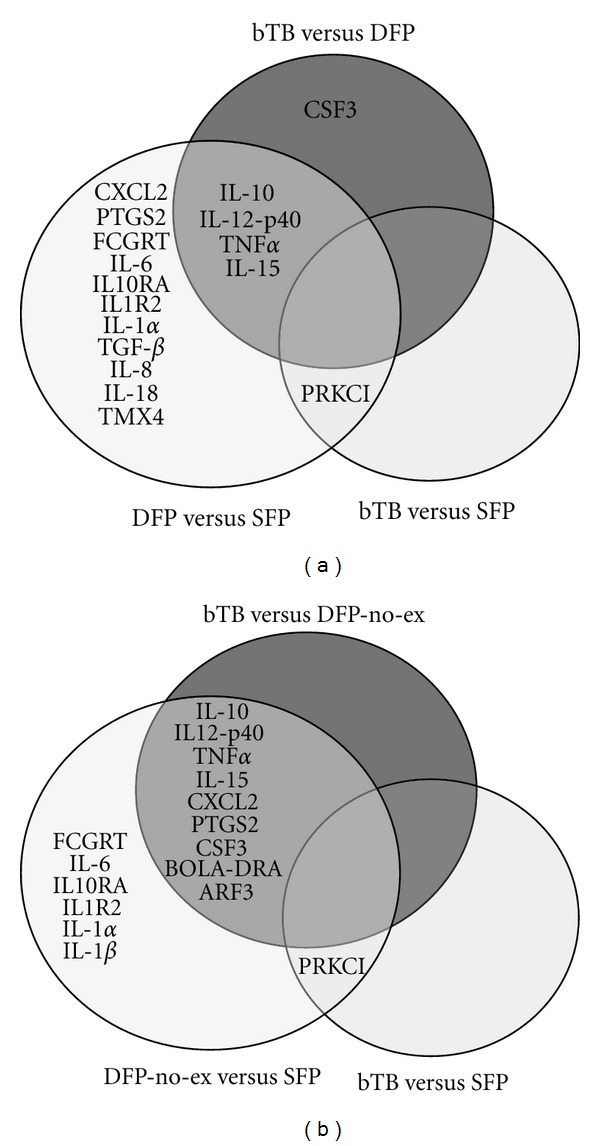
Venn diagrams showing the statistically significant (adj *P* ≤ 0.05) differentially expressed genes that were unique to or common among (a) single antemortem test-false positive (SFP), double antemortem test-false positive (DFP), and bTB-infected (bTB) groups of cattle in initial analysis of 30 cattle and (b) single antemortem test-false positive (SFP), double antemortem test-false positive non-bTB-exposed (DFP-non-ex), and bTB-infected (bTB) groups of cattle after removal of bTB-exposed cattle from the DFP group. The gene expression levels were determined by qPCR assay, where each animal was calibrated relative to the reference pool of RNA from healthy cattle; differential expression between 2 groups of cattle (i.e., *X* versus *Y*) was determined using ANOVA analysis.

The data from qPCR assays were analyzed at the individual animal level using the ANOVA test to identify genes that could significantly differentiate individual cattle within a group from cattle in the other groups. Of the 33 genes selected for qPCR assay, 17 were found to have differential power at adj *P* ≤ 0.05. The expression levels of 16 of those genes could be used to differentiate DFP cattle from SFP cattle (Figure 4(a)). Only 5 genes differentiated bTB cattle from DFP cattle, and only 1 gene significantly differentiated bTB cattle from SFP cattle. These results suggest that the gene expression profile of the SFP cattle was more similar to that of the bTB cattle than the DFP cattle. This finding was unexpected, because it was anticipated that the SFP and the DFP groups of cattle would be closer in expression profile to each other than to the bTB cattle.

All 10 SFP cattle and 4 of 10 cattle in the DFP group originated from bTB-positive herds and may have been exposed with *M. bovis.* Exposure with *M. bovis* might have influenced the gene expression profiles. The remaining 6 cattle in DFP group that did not have a history of bTB exposure were designated as double antemortem test-false positive non-bTB exposed (DFP-non-ex, *n* = 6), and the gene expression data from the qPCR assays were reanalyzed for this new group. Overall, removal of bTB-exposed cattle from the DFP-non-ex group did not have a statistically significant (adj *P* ≤ 0.05) effect on the mean expression values for most of the genes previously identified. However, the mean expression levels of 8 down regulated genes in the DFP-non-ex group were further down regulated by >2-fold change. Those genes are CXCL2, GCP2, IL-10, IL-1*α*, IL-1R2, IL-6, IL-8, and PTGS2. In addition, the mean expression levels of IFN-*γ* and PPP2R5B were no longer statistically significant for the DFP-non-ex group. The loss of statistical significance was attributed to the wide range of expression levels for those genes among the cattle and the reduction in the size of the group from 10 to 6 cattle.

Removal of the bTB-exposed cattle from the DFP group also affected the analysis at the individual animal level, using the ANOVA test. The differential power of 4 genes (TGF-*β*, IL-8, IL-18, and TMX4) was no longer statistically significant, but 2 additional genes (BOLA-DRA and ARF3) had statistically significant differential power among 2 or more groups of cattle. Thus, altered expression level of 15 genes was deemed significant (adj *P* ≤ 0.05) for differentiation of the 3 groups of cattle (Figure 4(b)). After removal of the bTB-exposed cattle, the number of genes that could be used to differentiate cattle in the DFP-non-ex group from cattle in the bTB group increased from 5 to 8 (Table 2).

## 4. Discussion

Currently, the OIE-approved bTB tests for international trade of cattle are the tuberculin skin tests (TSTs), which are based on a physically measurable cell-mediated inflammatory response against tuberculin antigen injected into either the skin of the neck or the caudal fold of the tail, and the IFN-*γ* ELISA assay, which measures IFN-*γ* secreted into plasma after stimulation of whole blood with tuberculin antigen [2, 41]. The TSTs are most commonly used to screen for bTB and normally are effective for control of bTB. However, limitations in sensitivity and specificity of TST have long been recognized [2, 42]. To increase diagnostic sensitivity, the IFN-*γ* ELISA assay is used in some countries in parallel with, or sequential to, various applications of the TST [43, 44]. Regardless of testing schemes employed, false positive and false negative test results remain an issue for bTB control programs. In Michigan, the current rate of bTB-infection is extremely low, which leads to far more antemortem test-false positive cattle being culled as bTB suspects than the number of bTB infected cattle identified at postmortem examination. This has driven our interest in comparing the gene expression profiles of bTB-positive and bTB antemortem test-false positive cattle. We hypothesized that altered transcription levels of select genes could discriminate between cattle infected with bTB and cattle that were antemortem test-false positive using currently approved diagnostic assays.

To test our hypothesis, mRNA expression levels were evaluated by microarray analysis that made use of a common reference design. The common reference used in the current study was a pool of RNA extracted from PBMC of healthy cattle after samples of whole blood from those cattle were stimulated for 4 hours with bPPD. The methods for antigen-stimulation and for RNA extraction from PBMC of healthy cattle were identical to those used for the 3 study groups of SFP, DFP, and bTB cattle. Previous studies have shown that bPPD stimulation of PBMC from healthy cattle will induce altered gene expression [16, 22]. By normalizing each microarray with a pool of RNA from antigen stimulated cells obtained from healthy cattle, it was hoped that any changes in gene expression that were due to nonspecific stimulation caused by bPPD would be filtered out.

After 2–4 hours of antigen stimulation, comparable microarray studies on cattle infected with MAP [45] or bTB [16, 22] have shown rapid changes in gene expression profiles of PBMC. Importantly, a marked increase was reported in the number of differentially expressed genes in bTB-positive cattle compared with TST-negative cattle following a 3-hour stimulation of whole blood with bPPD [16]. In the current study, we also found rapid changes in gene expression in bTB-positive cattle (bTB group) and in antemortem test-false positive cattle (SFP and DFP groups), after stimulation of blood with bPPD for 4 hours. At 0.01 level of significance, the number of genes showing altered expression was similar among the SFP (*n* = 51) and DFP groups (*n* = 60). In comparison, the numbers of genes showing altered expression in the bTB group were substantially less (*n* = 20). Most genes that showed altered expression were unique to the individual groups of cattle, and only a few genes were shared among 2 or more groups of cattle.

Although microarray hybridization analyses are useful as a general screening tool for identifying genes that show altered expression [13, 23], qPCR is accepted as the more sensitive and accurate assay for quantifying differential gene expression [46, 47]. Thus, qPCR was used to validate altered expression levels for select genes. The qPCR assays conducted in the current study confirmed that there were differences in gene expression between the SFP, DFP, and bTB groups of cattle (Figure 3). Compared with the reference pool of RNA, the expression of many genes was decreased at 4 hours after stimulation, especially in the DFP group of cattle. This finding was consistent with other studies that report a temporal decrease in level of gene expression following antigen stimulation of PBMC [22, 45]. The greatest increase in gene expression was observed for the cytokine IFN-*γ*, an essential event for the whole blood IFN-*γ* ELISA assay for bTB [48]. However, the IFN-*γ* gene was not useful for separating infected from noninfected cattle in the current study because the expression levels for that gene varied considerably among animals both within and between groups. Similarly, variability was observed in the amount of IFN-*γ* detected using the whole blood IFN-*γ* ELISA assay on samples of blood obtained from cattle in the current study (data not shown). Furthermore, the levels of altered expression of mRNA did not correlate with optical density readings from plasma in the IFN-*γ* ELISA assay.

The gene expression data from qPCR assays were analyzed to identify gene targets that might differentiate antemortem test-false positive cattle from the true bTB-infected cattle. Differentiation of the DFP cattle from the bTB group and the SFP groups of cattle was possible based on the expression levels of 5 and 16 genes, respectively (Figure 4(a)). However, only one of the genes subjected to qPCR assay could differentiate the bTB group of cattle from the SFP group. The origin of the cattle in the SFP group suggested a possible explanation for that finding. All of the cattle in the SFP group were exposed to *M. bovis* infected cohorts; thus, it was possible that some of the cattle in the SFP group had been infected with *M. bovis*. Similarly, a few of the cattle in the DFP group were exposed to* M. bovis* infected cohorts. We could not reevaluate the SFP group; however, we could reevaluate the DFP group by forming a new group of cattle (DFP-non-ex) that consisted of cattle with no known exposure to *M. bovis*. When the data from the qPCR were reanalyzed using the new groups of cattle, the list of genes that had statistically significant differential power to separate the DFP non-ex-group from the bTB group increased in number from 5 to 8 (Figure 4(b)).

The process of postmortem examination is not perfect; infections with *M. bovis* prior to lesion development may not be detected. It is thought that up to 30% of cattle in an infected herd can become infected with bTB [49]. Cattle with an effective innate immune response may clear an infection with* M. bovis.* In that event, those cattle might test positive by TST and/or IFN-*γ* ELISA assay but lack lesions at postmortem examination and be negative for *M. bovis* on bacterial culture [50]. Similarly, cattle in an early stage of infection with *M. bovis* may test positive by TST and/or IFN-*γ* ELISA assay but lack lesions at postmortem examination and be negative on cultures for *M. bovis* [51, 52]. Latent infection is known to occur in humans infected with *M. tuberculosis,* and it is believed that latent bTB infection can occur in cattle [53–56]. It is likely that some latently infected cattle would test positive by TST and/or IFN-*γ* ELISA assay but lack lesions at postmortem examination and be negative on cultures for *M. bovis* [53, 54]. Thus, failure to identify genes with altered expression that can differentiate bTB-infected cattle from antemortem test-false positive-bTB-exposed cattle may have been due to use of “non-infected” cattle that were, or had been, infected with bTB.

The current study examined altered expression of genes in PBMC at 4 hours following stimulation with bPPD. The gene expression profiles of the DFP-non-ex group of cattle were clearly different than those of the bTB group of cattle. That finding provides support for the hypothesis that detection of altered expression of a few genes could be used to differentiate bTB-infected cattle and antemortem test-false positive cattle from bTB-free herds. Temporal studies that used antigen stimulation of PBMC from cattle infected with bTB have shown that there is a rapid and transient burst of gene expression that occurs within hours of antigen stimulation. A second burst of altered gene expression occurs at 12 to 24 hours after stimulation [16, 22, 23]. The current study tried to capitalize on the early burst of altered gene expression. We recently conducted a similar study analyzing gene expression profiles after overnight stimulation of PBMC with antigen. As expected, it was observed that many of the genes that showed altered expression after 4 hours of antigen stimulation in the current study did not show altered expression after overnight incubation with antigen. Instead a new set of genes with altered expression were identified that may be evaluated for use as molecular markers for segregation of bTB-infected cattle from noninfected cattle. The results of the current study indicate that monitoring altered expression of genes with differential power has potential to separate bTB-infected cattle from antemortem test-false positive cattle in bTB-free herds. However, further studies are needed to evaluate the gene expression profiles of the antemortem test-false positive cattle from both low-risk herds for bTB exposure and cattle from bTB-infected herds.

## 5. Conclusions

The results from differential gene expression analyses reported here clearly showed that gene expression profiles differed between the DFP-non-ex group of cattle and the bTB group of cattle. However, differentiating the bTB-infected cattle from the antemortem test-false positive cattle that had been exposed to bTB infected cattle in the field was problematic. Therefore, further work is needed to gain a better understanding of the distinct differences in gene expression profiles of these cattle. The results from this study are encouraging for use of altered gene expression profiles in the development of ancillary tests for bTB that can improve the diagnostic process and reduce the unnecessary destruction of antemortem test-false positive cattle from bTB-free herds.

## Supplementary Material

Supplementary Table S1: Genes from microarray analysis that showed statistically significant (*p* ≤ 0.01) differential expression in one or more of the single test-false positive (SFP), double test-false positive (DFP) and bTB infected (bTB) groups of cattle after a 4 hour stimulation of whole blood with tuberculin.Differential expression is shown here as log fold change.
Supplementary Table S2: Genes from microarray analysis that showed statistically significant (*p* ≤ 0.01) differential power between the single test-false positive (SFP), double test-false positive (DFP) and bTB infected (bTB) groups of cattle after a 4 hour stimulation of whole blood with tuberculin. Differential expression in log fold change was computed between the bTB and the DFP groups (bTB versus DFP), the bTB and the SFP groups (bTB versus SFP), and the DFP and the SFP groups (DFP versus SFP).

## Figures and Tables

**Table 1:** Genes selected for qPCR analysis, the nucleic acid sequence and concentration (nM) of PCR primers for those genes (forward primer (F) and reverse primer (R)), the PCR efficiency (E), and the PCR amplicon sizes (bp).

	Gene symbol	Gene name	Primer (5′-3′)	Primer conc. (nM)	PCR eff. (E)	Amplicon size (bp)
#	ARF3	ADP-ribosylation factor 3	F: TTGCCTAATGCCATGAATGC R: CACAGGTGGCCTGAATGTA	300	1.817	91
#	BOLA-DMA	Major histocompatibility complex, class II, DM *α*-chain	F: TTGTTGGCTTGGTCCTCTTC R: ACACCTCCTGCTTGGATGG	300	1.975	105
#	BOTL08_C07	Unknown	F: ATCACTTCCCGCCTCCTTAG R: AGGCAGGTGACCAAGGAAAC	600	1.925	92
#	CXCL2	C-X-C ligand 2 (GRO-alpha)	F: AACAAGGCTAGTGCCAACTG R: CCACTGAGGCTGCTGGAG	300	1.912	68
#	DDX5	DEAD (Asp-Glu-Ala-Asp) box polypeptide 5	F: AGAGATCTGGTGGGTAGCTTTA R: ACCCTATCCTCTCCTTGCAAAC	300	1.917	79
#	IL-4	Interleukin-4	F: GCCACACGTGCTTGAACAAA R: TGCTTGCCAAGCTGTTGAGA	450	1.910	63
#	LTBR	Lymphotoxin beta receptor (TNFR superfamily member3)	F: CCGGAGTGACGAGGAAGAC R: CAAAACTCGCCCTTATACCTTG	450	1.859	104
#	PPP2R5B	Protein phosphatase 2, regulatory subunit B′, beta isoform	F: GTGGTCCTGGCAACAGAAC R: CTGGAGCCCAGCTTTGTG	300	1.895	110
#	PRKCI	Protein kinase C, iota	F: CAAGGACCCAAAGGAACGATT R: ACCACCTGCTTTTGCTCCAT	300	1.897	114
#	PTGS2	Prostaglandin-endoperoxide synthase 2(cyclooxygenase-2)	F: CGACACCAAGAACGTATTCCTA R: GAGATGTGGAAAAGAAGCATTG	300	1.930	105
*	BOLA-DRA	MHC class II DR alpha	F: GCTCTGGTGGGCATCATTG R: CCTCGGCGTTCAACGGTG	300	1.910	77
*	TPRA1	Transmembrane protein, adipocyte associated 1	F: GTGCGCAGACATCATTGAG R: GGCGCAAAGAAGCTGAAG	450	1.974	72
*	TRIM13	Tripartite motif-containing 13	F: CTGGCACGTTCATTAGCAAG R: GGCCAAGCAGAATGACCAC	300	1.962	69
*	FCGRT	Fc fragment of IgG, receptor, transporter, alpha	F: GGCCCGAATCGTTGTGTT R: GAAGCCCAAGGCTTACACC	450	1.822	81
*	TMX4	Thioredoxin-related transmembrane protein 4	F: ACCTTGACTTGTGCTCACTT R: TGGAGGTACCACTGGAACTG	300	1.993	85
*	BOTL11_A05	Unknown	F: CACACTCTATGGCGCAAATC R: CCCTGGACCACCACCTCTA	300	1.903	75
*	RPL19	Ribosomal protein L19	F: GGCTCCAGGCCAAGAAAG R: AATTGCCGAGGCCACTATG	300	1.972	106
§	CSF3	Colony stimulating factor 3 (granulocyte)	F: CTGGGTGAGACTGGGAAATG R: TCTCTCACACCCCGTCACA	300	1.959	62
§	GCP2	Granulocyte chemotactic protein 2 (CXCL6)	F: CATTGGAATGCTGTATATGGAGAT R: TCTTCCAAAGGTCAAGAGTAAGA	300	1.874	122
§	IL-10	Interleukin-10	F: CTTGTCGGAAATGATCCAGTTTT R: TCAGGC CCGTGG TTCTCA	300	1.948	66
§	IL-10RA	Interleukin-10 receptor A	F: GTCACCCTGCCACTGATCAC R: GGCAGCGTGCAGCTGAAATC	300	1.828	84
§	IL-6	Interleukin-6	F: GGCTCCCATGATTGTGGTAGTT R: GCCCAGTGGACAGGTTTCTG	300	1.873	64
§	IL-12p40	Interleukin-12, p40 subunit	F: CAAACCAGACCCACCCAAGA R: GACCTCCACCTGCCGAGAA	300	1.896	64
§	IL-15	Interleukin-15	F: GGCTGGCATTCATGTCTTCA R: CATACT GCCAGT TTGCTTCTGTTT	300	1.850	74
§	IL-18	Interleukin-18	F: GAAAATGATGAAGACCTGGAATCA R: ACTTGGTCATTCAAATTTCGTATGA	300	1.896	84
§	IL-1b	Interleukin-1 beta	F: AAGCAGGCGCATCTGTGAA R: ATGGCACTCTAACCCGGAAA	450	1.915	70
§	IL1R2	Interleukin-1 receptor 2	F: ATACCTGTGCCATGACGTATGC R: CGGAGTTTGATATTCCTGGTGAT	300	1.923	67
§	IL2	Interleukin-2	F: TGATGCAACAGTAAACGCTGTAG R: GAGAGGCACTTAGTGATCAAGTC	450	1.928	95
§	IL-1*α*	Interleukin-1 alpha	F: TTGGTGCACATGGCAAGTG R: GCACAGTCAAGGCTATTTTTCCA	450	1.948	72
§	IL-8	Interleukin-8	F: GGAAAAGTGGGTGCAGAAGGT R: GGTGGTTTTTTCTTTTTCATGGA	100	1.888	80
§	INF-*γ*	Interferon, gamma	F: TGGCATGTCAGACAGCACTTG R: CCTGAAGCGCCAGGTATAAGG	450	1.932	96
§	TGF*β*	Transforming growth factor, beta	F: CTGAGCCAGAGGCGGACTAC R: TGCCGTATTCCACCATTAGCA	300	1.897	63
§	TNF*α*	Tumor necrosis factor, alpha	F: TCTACCAGGGAGGAGTCTTCCA R: GTCCGGCAGGTTGATCTCA	300	1.871	68
	SDHA	Succinate dehydrogenase complex subunit A	F: CCACGCCAGGGAGGACTTC R: CGTAGGAGAGCGTGTGCTTC	300	1.879	116

^#^Genes that showed substantial altered expression within a group of cattle in microarray studies.

*Genes that had differential power between groups of cattle using microarray expression data analyzed with MAANOVA.

^§^Genes that were selected from the literature as being relevant to the bTB infection.

**Table 2:** Genes that showed differential power (adj *P* ≤ 0.05) between cattle that were double antemortem test-false positive with no bTB-exposure history (DFP-non-ex) and cattle that were bTB infected (bTB), as determined by qPCR analysis. The differential expression level (Δlog⁡_2_⁡FC) of the bTB and DFP-non-ex groups of cattle (bTB minus DFP-non-ex) was determined using ANOVA analysis.

Gene	Δlog⁡_2_⁡FC (bTB versus DFP-non-ex)	adj *P *
IL-10	1.852	0.0314
IL12-p40	1.975	0.0267
TNF*α*	1.809	0.0217
PTGS2	3.413	0.0183
CSF3	2.199	0.0175
CXCL2	2.498	0.0175
BOLA-DRA	1.010	0.0175
ARF3	0.736	0.0175
